# Spatiotemporal Dynamics of Coastal Viral Community Structure and Potential Biogeochemical Roles Affected by an Ulva prolifera Green Tide

**DOI:** 10.1128/msystems.01211-22

**Published:** 2023-02-23

**Authors:** Meiaoxue Han, Jianhua Sun, Qingwei Yang, Yantao Liang, Yong Jiang, Chen Gao, Chengxiang Gu, Qian Liu, Xuechao Chen, Gang Liu, Hongbing Shao, Cui Guo, Hui He, Hualong Wang, Yeong Yik Sung, Wen Jye Mok, Li Lian Wong, Zongling Wang, Andrew McMinn, Min Wang

**Affiliations:** a College of Marine Life Sciences, Key Lab of Polar Oceanography and Global Ocean Change, Institute of Evolution and Marine Biodiversity, and Frontiers Science Center for Deep Ocean Multispheres and Earth System, Ocean University of China, Qingdao, China; b University Malaysia Terengganu-Ocean University of China Joint Centre for Marine Studies, Qingdao, China; c Institute of Marine Biotechnology, University Malaysia Terengganu, Kuala Nerus, Malaysia; d Key Laboratory of Marine Eco-Environmental Science and Technology, First Institute of Oceanography, Ministry Natural Resources, Qingdao, China; e Institute for Marine and Antarctic Studies, University of Tasmania, Hobart, Tasmania, Australia; f Haide College, Ocean University of China, Qingdao, China; g Affiliated Hospital of Qingdao University, Qingdao, China; University of Massachusetts Amherst

**Keywords:** virome, *Ulva prolifera* green tide, spatiotemporal dynamics, viral auxiliary metabolic genes, coastal waters of Yellow Sea

## Abstract

The world’s largest macroalgal green tide, caused by Ulva prolifera, has resulted in serious consequences for coastal waters of the Yellow Sea, China. Although viruses are considered to be one of the key factors in controlling microalgal bloom demise, understanding of the relationship between viral communities and the macroalgal green tide is still poor. Here, a Qingdao coastal virome (QDCV) time-series data set was constructed based on the metagenomic analysis of 17 DNA viromes along three coastal stations of the Yellow Sea, covering different stages of the green tide from Julian days 165 to 271. A total of 40,076 viral contigs were detected and clustered into 28,058 viral operational taxonomic units (vOTUs). About 84% of the vOTUs could not be classified, and 62% separated from vOTUs in other ecosystems. Green tides significantly influenced the spatiotemporal dynamics of the viral community structure, diversity, and potential functions. For the classified vOTUs, the relative abundance of Pelagibacter phages declined with the arrival of the bloom and rebounded after the bloom, while Synechococcus and Roseobacter phages increased, although with a time lag from the peak of their hosts. More than 80% of the vOTUs reached peaks in abundance at different specific stages, and the viral peaks were correlated with specific hosts at different stages of the green tide. Most of the viral auxiliary metabolic genes (AMGs) were associated with carbon and sulfur metabolism and showed spatiotemporal dynamics relating to the degradation of the large amount of organic matter released by the green tide.

**IMPORTANCE** To the best of our knowledge, this study is the first to investigate the responses of viruses to the world’s largest macroalgal green tide. It revealed the spatiotemporal dynamics of the unique viral assemblages and auxiliary metabolic genes (AMGs) following the variation and degradation of Ulva prolifera. These findings demonstrate a tight coupling between viral assemblages, and prokaryotic and eukaryotic abundances were influenced by the green tide.

## INTRODUCTION

Massive green tides, induced by climate warming and eutrophication, occur frequently worldwide (1). The largest causative green tide organism is the macroalgae Ulva prolifera, which has occurred in successive years in the Yellow Sea, China, since 2007. The green tide has caused complex and profound effects on marine ecosystems and has had a serious negative economic impact (2). On the positive side, however, U. prolifera assimilates a large amount of inorganic carbon in the early and middle stages of the green tides, increasing the pH of the seawater and subsequently sequestering a large amount of CO_2_ from the Yellow Sea. However, during the terminal phases of the green tides, millions of tons of macroalgal biomass are advected adjacent to coastal cities along the Yellow Sea, causing great pressure on the coastal environment. Of these cities, Qingdao, with a population of 10 million, has been the most affected. The decomposition of the U. prolifera biomass consumes a large amount of oxygen and releases hydrogen sulfide, causing coastal hypoxia and acidification (3). At the same time, millions of tons of U. prolifera gradually sink to the bottom, increasing the vertical export of organic matter to the sediment (4). The dynamic progression of the U. prolifera green tide triggers significant and often successive shifts in microbial community structure (5). For example, the strands of U. prolifera are commonly populated by abundant epiphytic heterotrophic diazotrophs, which contribute to the nitrogen demand of U. prolifera during the blooms (6, 7). A close relative, Ulva linza, depends on the regulation of the key microbial group Cytophaga-Flexibacter-Bacteroides to ensure the normal growth of its strands (8). Dynamic changes in dissolved organic carbon (DOC) concentration and a significant succession in the bacterial community structure and function accompany the long-term degradation of U. prolifera (9, 10).

Viruses are the most abundant and diverse biological entities in the sea, significantly influencing microbial abundance and community structure through viral lysis (11, 12), mediation of marine biogeochemical cycles (12, 13), and manipulation of host cell metabolism through the expression of viral-encoded auxiliary metabolic genes (AMGs) (14). Viruses also play an important role in algal blooms (15, 16). Algal viruses can regulate the dynamics of microalgal blooms directly through viral lysis, a process called the “viral shunt,” and influencing host genetic composition (17, 18). They can also remodel the metabolism of the host cells, including the pathways of glycolytic fluxes, fatty acid synthesis, and viral-derived glycosphingolipids (19–21). Viral abundance also shows a significant positive relationship with bacterial populations, which indirectly influence the algal biomass through nutrient remineralization of the viral-induced release of dissolved organic matter (DOM) (22). Recently, it has been proposed that algal viruses can enhance the production of transparent exopolymer particles (TEPs) and aggregates and the vertical exportation of DOM, also part of the viral shuttle (14, 23, 24). Viral infection is considered to be one of the major factors controlling algal succession and the collapse of microalgal blooms (14, 17).

Although viral dynamics are closely related to the formation and progression of algal blooms, our understanding of their roles in green tides is still limited. In this study, using metagenomics, we analyzed the spatiotemporal dynamic, community structure, and AMGs of metagenomic-assembled viral contigs and their relationship with host cells and environmental parameters at three stations in the coastal waters of Qingdao, during and after a U. prolifera green tide, from Julian days 165 (14 June) to 271 (28 September) in 2017. This study provides the first glimpse of the viral assemblages and potential biogeochemical roles during an outbreak and demise of a green tide.

## RESULTS

### Environmental parameters, overview of the Qingdao coastal virome (QDCV) data set, and its relationships with environmental factors and other environmental viromes.

**FIG 1:**
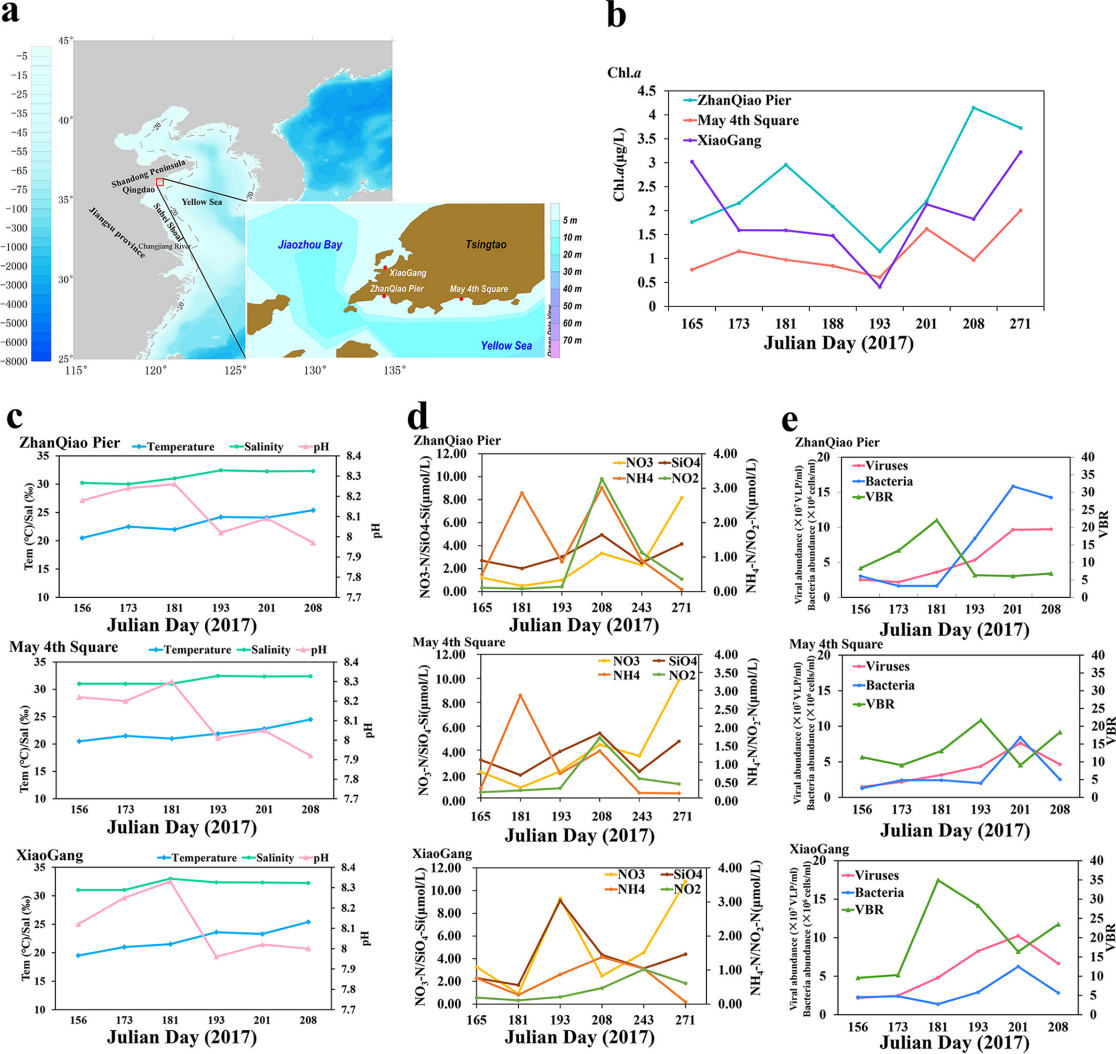
(a) Map of sampling stations around coastal waters of Qingdao with the influences of macroalgae U. prolifera green tide. Topography data of the map are derived from Etopo2 data set. The red dots represented the three sampling stations. The XiaoGang (XG) station is located in the Jiaozhou Bay. The ZhanQiao Pier (ZQ) and May 4th Square (MS) stations are located in the northern coastal waters of Yellow Sea, where was the primary green tide area of macroalgae U. prolifera. (b to e) Physicochemical characteristics. (b) chl. *a*, (c) Temperature, salinity, and pH. (d) Nutrients. (e) Bacterial and viral abundance changed during different stage of U. prolifera bloom. Chl.a, chlorophyll a; Sal, salinity; Tem, temperature; VBR, virus-to-bacteria ratio.

The physicochemical spatiotemporal dynamics of the sampling sites are shown in Fig. 1. In general, the concentration of chlorophyll *a* (chl. *a*) was higher after the blooms (on Julian days 243 and 271) than during the blooms (on days 165, 181, 193, and 208), with the lowest value on day 193 and then gradually increasing at all three stations (Fig. 1b; Table S1). This is counter to what might be expected and may be due to the growth of some microalgae after the green tide (25, 26), resulting in an increase in chlorophyll concentration in seawater. The temperature and salinity gradually increased with the bloom, while pH values increased after the arrival of bloom (on day 165) and then gradually decreased starting on day 181 (Fig. 1c; Table S1). There was a similar temporal pattern in nutrient concentrations at all three stations; peak values of NH_4_, SiO_3_, and NO_2_ were found on day 208. Unlike other nutrients, nitrate also showed a clear upward trend after the bloom (on day 271). Viral and bacterial abundances increased several times, from day 165 to peak values on day 201 and then decreased on day 208. The virus-to-bacteria ratio (VBR) increased from day 165 to day 193 and then decreased to minimum values on day 201 (Fig. 1e).

10.1128/msystems.01211-22.6 TABLE S1 Environmental parameters. Download Table S1, XLSX file, 0.01 MB. Copyright © 2023 Han et al. 2023 Han et al. https://creativecommons.org/licenses/by/4.0/ This content is distributed under the terms of the Creative Commons Attribution 4.0 International license.

The Qingdao coastal virome (QDCV) data set contained 17 DNA virome libraries with a total of 176.85 Gb of sequence data. A total of 236,478 contigs longer than 3 kb were assembled. Combined with the prediction outputs of VirSorter, VirFinder, and Contig Annotation Tool (CAT), a total of 40,076 viral contigs were detected and clustered into 28,058 viral operational taxonomic units (vOTUs, Table S2). Most (84.8%) vOTUs were shared among the three stations, and only 6.2% of vOTUs (1.7% in ZhanQiao [ZQ], 0.9% in May 4th Square [MS], and 3.6% in XiaoGang [XG]) were at a single station (Fig. S1b). However, the vOTUs of the QDCV changed with the arrival (on day 165) and termination (on day 208) of the U. prolifera bloom (Fig. S1a), and the viral diversity, Shannon index (Shannon’s *H*) and Peilou’s *J*, increased from during the bloom (on days 165, 181, 193, and 208) to after the bloom (on days 243 and 271) (Fig. S1d).

10.1128/msystems.01211-22.1 FIG S1 (a) UpSet plot visualizing the intersecting sets of viral operational taxonomic units (vOTUs) during the different stages of U. prolifera bloom at the three stations. (b) Venn plot of shared and specific vOTUs among the three stations. (c) Principal-component analysis (PCA) of the virome samples at three stations during the different stages of U. prolifera bloom. (d) Variations of α-diversity indexes of three stations during the different stages of U. prolifera bloom. Download FIG S1, TIF file, 1.5 MB. Copyright © 2023 Han et al. 2023 Han et al. https://creativecommons.org/licenses/by/4.0/ This content is distributed under the terms of the Creative Commons Attribution 4.0 International license.

10.1128/msystems.01211-22.7 TABLE S2 Basic information about Qingdao coastal virome (QDCV). Download Table S2, XLSX file, 0.01 MB. Copyright © 2023 Han et al. 2023 Han et al. https://creativecommons.org/licenses/by/4.0/ This content is distributed under the terms of the Creative Commons Attribution 4.0 International license.

**FIG 2:**
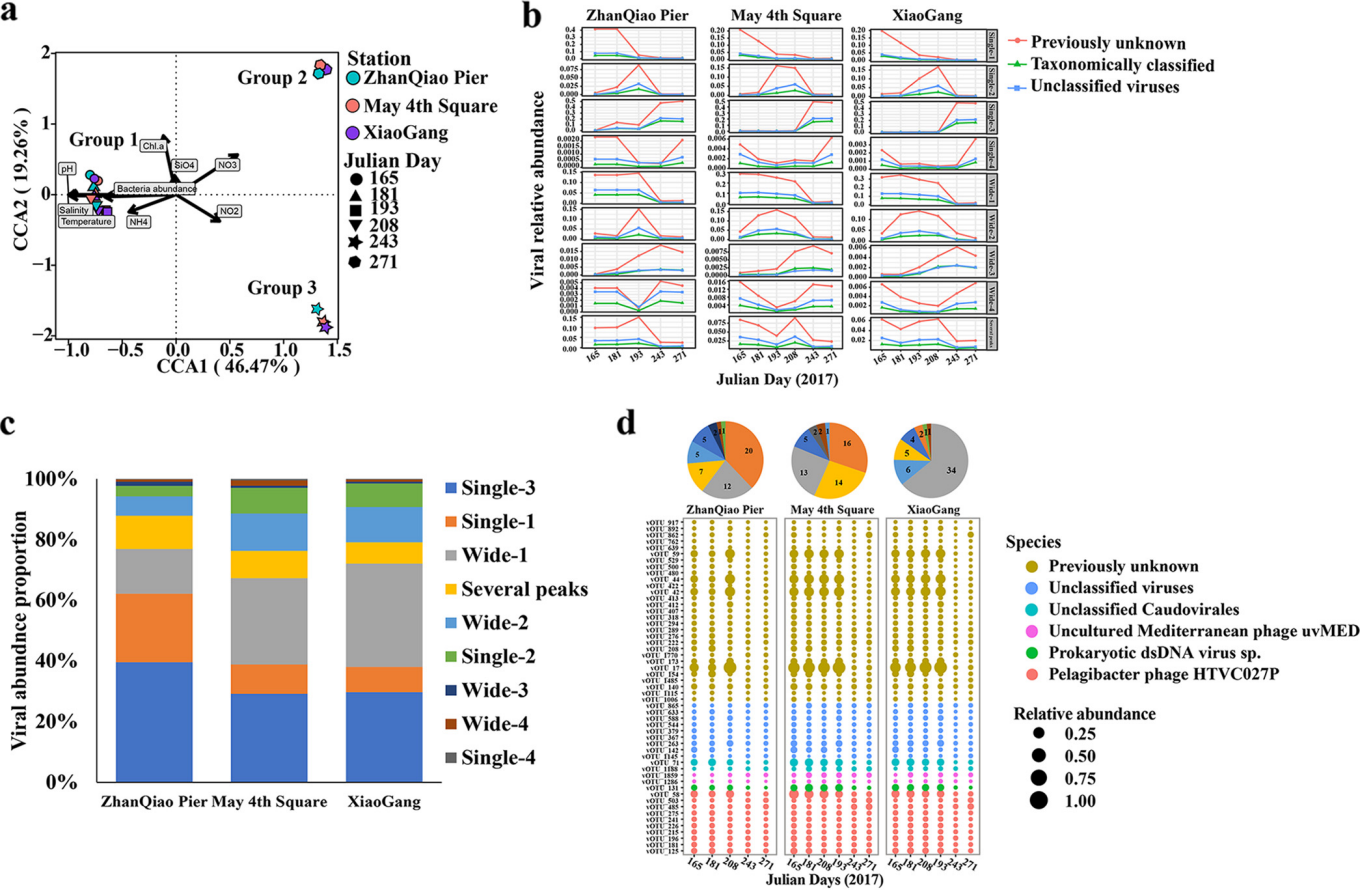
The diversity and succession of viral operational taxonomic units (vOTUs) in the Qingdao coastal virome (QDCV) data set. (a) Canonical correspondence analysis (CCA) of the relationship between vOTUs and environmental factors. Different colors and shapes represent different stations and time points, respectively. (b) Succession and dynamic changes of vOTUs abundance caused by the arrival of U. prolifera bloom. (c) Percentages of vOTUs of kinds of peak abundance in the three stations. (d) Relative abundance bubble plot of abundant vOTUs (vOTUs with the abundance greater than 1%). The sizes of the bubbles correspond to the relative abundance of vOTUs for each sample, and colors correspond to the viral taxa. dsDNA, double-stranded DNA. Chl.a, chlorophyll a.

The canonical correspondence analysis (CCA) between vOTUs and environmental factors showed that the virome samples could be divided into three groups (Fig. 2a), which is similar to the Principal-component analysis (PCA) results (Fig. S1c). The first two CCA axes explain 65.73% of the virome variations. The virome samples on days 165, 181, 193, and 208 were clustered together (group 1) and positively correlated with pH, temperature, salinity, bacterial abundance, and NH_4_ (Fig. 2a). The virome samples on day 243 (group 2) were positively correlated with NO_3_, and the virome samples on day 271 (group 3) were positively correlated with NO_2_. The virome samples from both days 243 and 271 were negatively related to pH, temperature, salinity, bacterial abundance, and NH_4_ (Fig. 2a).

**FIG 3:**
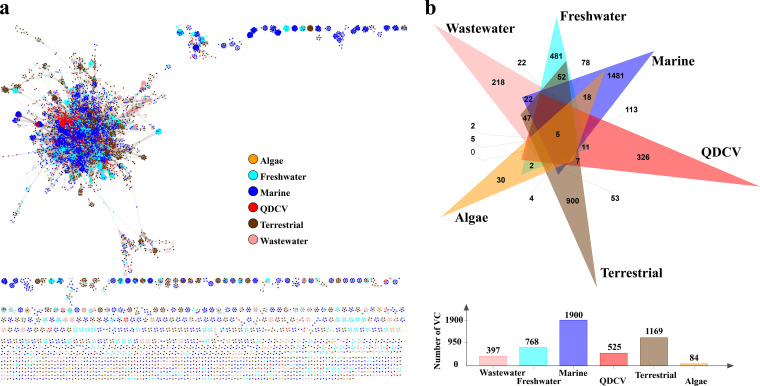
Comparison analysis between the Qingdao coastal virome (QDCV) data set and other environmental viromes. (a) A network of shared predicted protein content among viral operational taxonomic units (vOTUs, ≥10 kb) of QDCV influenced by the U. prolifera bloom (*n* = 4,633) and viral genomes from IMG/VR3.0 data set covering different habitats, including algae-host-associated, freshwater, marine, terrestrial, and wastewater. Nodes (circles) represent viral genomes and vOTUs, and the shared edges (lines) indicate shared protein content. Different colors represent different habitats. (b) Venn diagram of shared and specific viral clusters (VCs) among the six different environmental habitats.

More than 84% of the vOTUs could not be taxonomically classified at the family level (Fig. S2b), which was similar to the gene-sharing network analysis (Fig. S3a). From the gene-sharing network of the viral genomes (≥10 kbp) of the QDCV, which was influenced by the U. prolifera bloom, and the other environmental viromes from the Integrated Microbial Genomes/Viruses (IMG/VR) v3 data set, a total of 4,025 VCs were predicted, of which 525 VCs contained U. prolifera bloom-associated vOTUs in the QDCV data sets (Fig. 3a). Overall, most of the VCs in the QDCV (326 VCs, 62.1%) were endemic viruses, and only 199 (37.9%) were clustered with viral sequences from other habitats, including 113 VCs clustered with marine-derived vOTUs, 11 VCs clustered with freshwater-derived vOTUs, 7 VCs clustered with terrestrial-derived vOTUs, 2 VCs clustered with wastewater-derived vOTUs, and 5 VCs clustered with all other habitat-derived vOTUs (Fig. 3b).

10.1128/msystems.01211-22.2 FIG S2 (a) Histogram showing the proportion of lysogenic and lytic viruses. (b) Viral community structure at the family level in the three stations. Download FIG S2, TIF file, 0.9 MB. Copyright © 2023 Han et al. 2023 Han et al. https://creativecommons.org/licenses/by/4.0/ This content is distributed under the terms of the Creative Commons Attribution 4.0 International license.

10.1128/msystems.01211-22.3 FIG S3 Spatiotemporal variations of viral clusters (VCs). (a) A network of shared predicted protein content among viral operational taxonomic units (vOTUs) (≥10 kb) in the bloom of U. prolifera and RefSeq prokaryotic viral genomes. Nodes (circles) represent viral genomes and vOTUs, and the shared edges (lines) indicate shared protein content. Color represents the taxon annotation in RefSeq prokaryotic viral genomes and vOTUs in the bloom of U. prolifera. (b) Dynamic changes of VCs caused by the arrival of U. prolifera bloom. Download FIG S3, TIF file, 2.6 MB. Copyright © 2023 Han et al. 2023 Han et al. https://creativecommons.org/licenses/by/4.0/ This content is distributed under the terms of the Creative Commons Attribution 4.0 International license.

### Spatiotemporal dynamics of the viral community structure.

To draw a picture of the succession of viruses during the different stages of U. prolifera blooms at the three stations, the temporal dynamics of the peak abundance vOTUs are presented (Fig. 2b). Briefly, vOTUs with single peaks in relative abundance after the bloom (on days 243 and 271) and wide peaks during the bloom (on days 165, 173, 181, and 208) were the most abundant (Fig. 2b and c). In addition, 53 abundant vOTUs with an abundance greater than 1% were selected. Most (29 vOTUs) of the abundant vOTUs were previously unknown, 10 vOTUs were not classified. Of the classified vOTUs (15 vOTUs), 10 were classified as Pelagibacter phage HTVC027P (Fig. 2d).

**FIG 4:**
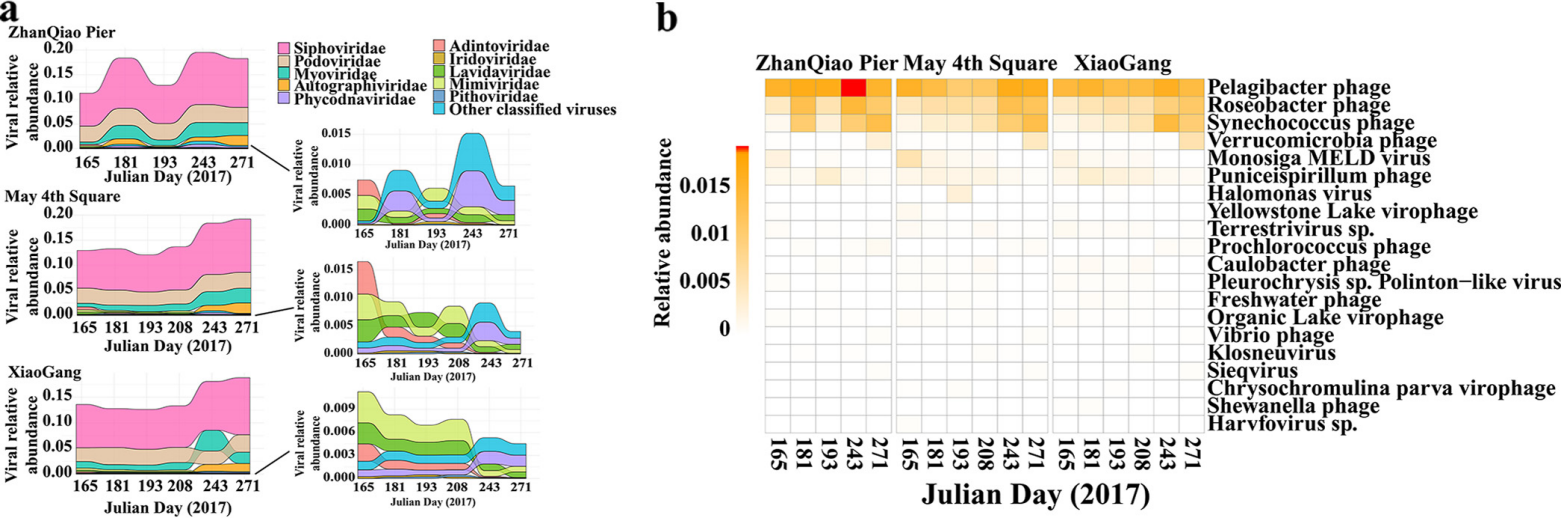
Spatiotemporal variations of classified viral operational taxonomic units (vOTUs) caused by the arrival of U. prolifera bloom. (a) Viral community structure at the family level during different stages of U. prolifera bloom in the three stations. (b) Heat map of the top 10 most abundant classified viruses based on the same level of host genera. Darker shades of red indicate higher relative abundance of that virus taxa.

For the 21% of vOTUs classified at the family level, most of them (ca. 94% to 95%) were classified into the *Caudovirales* order (Fig. S2b), followed by the families of nucleocytoplasmic large DNA viruses (NCDLV, including *Phycodnaviridae* and *Mimiviridae*) and virophages (*Lavidaviridae*) (Fig. S2b). Although the viral community structure of the three stations at the family level was similar, the relative abundance of different viral families varied after the arrival of U. prolifera (Fig. 4a). Viruses infecting Pelagibacter (SAR11) were the dominant group, but these decreased with the arrival of the bloom and then rebounded after the bloom (Fig. 4b). In contrast, the viruses infecting *Cyanobacteria* (Synechococcus phage and Prochlorococcus phage), *Verrucomicrobia* (*Verrucomicrobia* phage), Roseobacter (Roseobacter phage), and Vibrio (Vibrio phage) increased from during the bloom to after the bloom (Fig. 4b). In addition, *Phycodnaviridae* infecting eukaryotic algae increased after the bloom (Fig. 4a). *Lavidaviridae*, such as the Yellowstone Lake virophage, Organic Lake virophage, and Chrysochromulina parva virophage, increased during the bloom and decreased after the bloom. Viruses infecting protists, such as Terrestrivirus sp., Monosiga MELD virus 2, and Klosneuvirus, and infecting eukaryotic algae, such as Pleurochrysis sp. Polinton-like virus decreased with the arrival of the bloom (Fig. 4b). In addition, the spatiotemporal dynamics of vOTUs (Fig. 1c) was generally similar to the dynamics of VCs (Fig. S3b) and was tightly coupled to the dynamics of the community structure of the prokaryotes and eukaryotes (Fig. S4a and b), based on 16S and 18S rRNA genes (Qian Liu, Yuye Han, Yan Li, Mengyao Yang, Furong Cao, Min Wang, Yantao Liang, Hualong Wang, Cui Guo, Hui He, Hongbing Shao, Yong Jiang; submitted for publication).

10.1128/msystems.01211-22.4 FIG S4 Relationships between viruses and the hosts (bacteria and microbial eukaryotes). (a) The correlation network of viruses and hosts. The node size and node color represent the viral clusters (VCs) of the peak class of relative abundance or the OTU taxonomy of bacteria and microbial eukaryotes. The orange edge means that two nodes are positively correlated, while the purple edge means that two nodes are negatively correlated. (b) Heat map of the numbers of VCs significantly correlated with host bacteria and microbial eukaryotes in different observed cases. The shade of each square indicates numbers of VCs significantly correlated with their hosts. Orange and blue represent positive and negative correlations between VCs and their hosts, respectively. Download FIG S4, TIF file, 2.9 MB. Copyright © 2023 Han et al. 2023 Han et al. https://creativecommons.org/licenses/by/4.0/ This content is distributed under the terms of the Creative Commons Attribution 4.0 International license.

### Host prediction and lineage-specific virus-host relationships.

**FIG 5:**
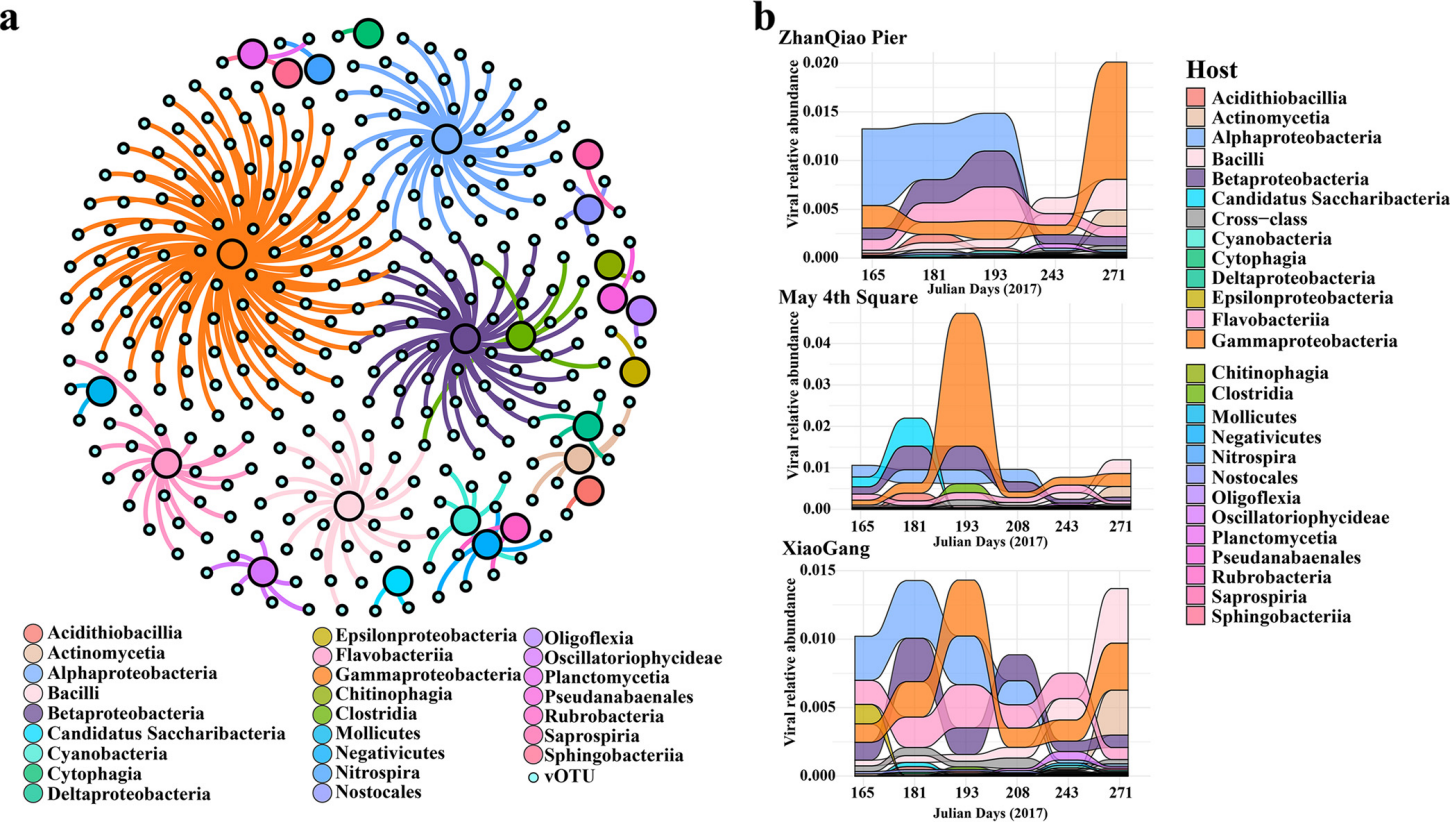
Virus-host linkages. (a) Network of putative virus-host linkages for viral operational taxonomic units (vOTUs, ≥5 kb) against bacterial NCBI RefSeq. Big circles represent different classes of prokaryotes, and small circles represent vOTUs of the Qingdao coastal virome (QDCV) data set. (b) Sandy plots of vOTUs relative abundance change during the different stages of U. prolifera bloom (vOTUs grouped by predicted host taxonomy).

Using the sequence similarity, tRNA sequences and CRISPR spacers, putative hosts were predicted for 278 of the 12,768 QDCV vOTUs (≥5 kb; Fig. 5a). Most predicted vOTUs had narrow host ranges, with only 12 potentially exhibiting a broader host range across several classes. Predicted prokaryotic hosts spanned 25 bacterial classes, with Gammaproteobacteria (33.1% of virus-host pairs) and Alphaproteobacteria (13.3%) as the most frequently predicted (Fig. 5a). The relative abundance of vOTUs linking to Alphaproteobacteria decreased from arrival of the bloom (on day 165) to after the bloom (on day 271) at all three stations, whereas the vOTUs linking to Gammaproteobacteria increased with the arrival of the bloom (on day 165) and reached peaks 2 months after the bloom (on day 271) at ZQ station and on day 193 at the MS and XG stations, respectively (Fig. 5b).

### Spatiotemporal dynamics of AMGs and relationships with environmental variables.

**FIG 6:**
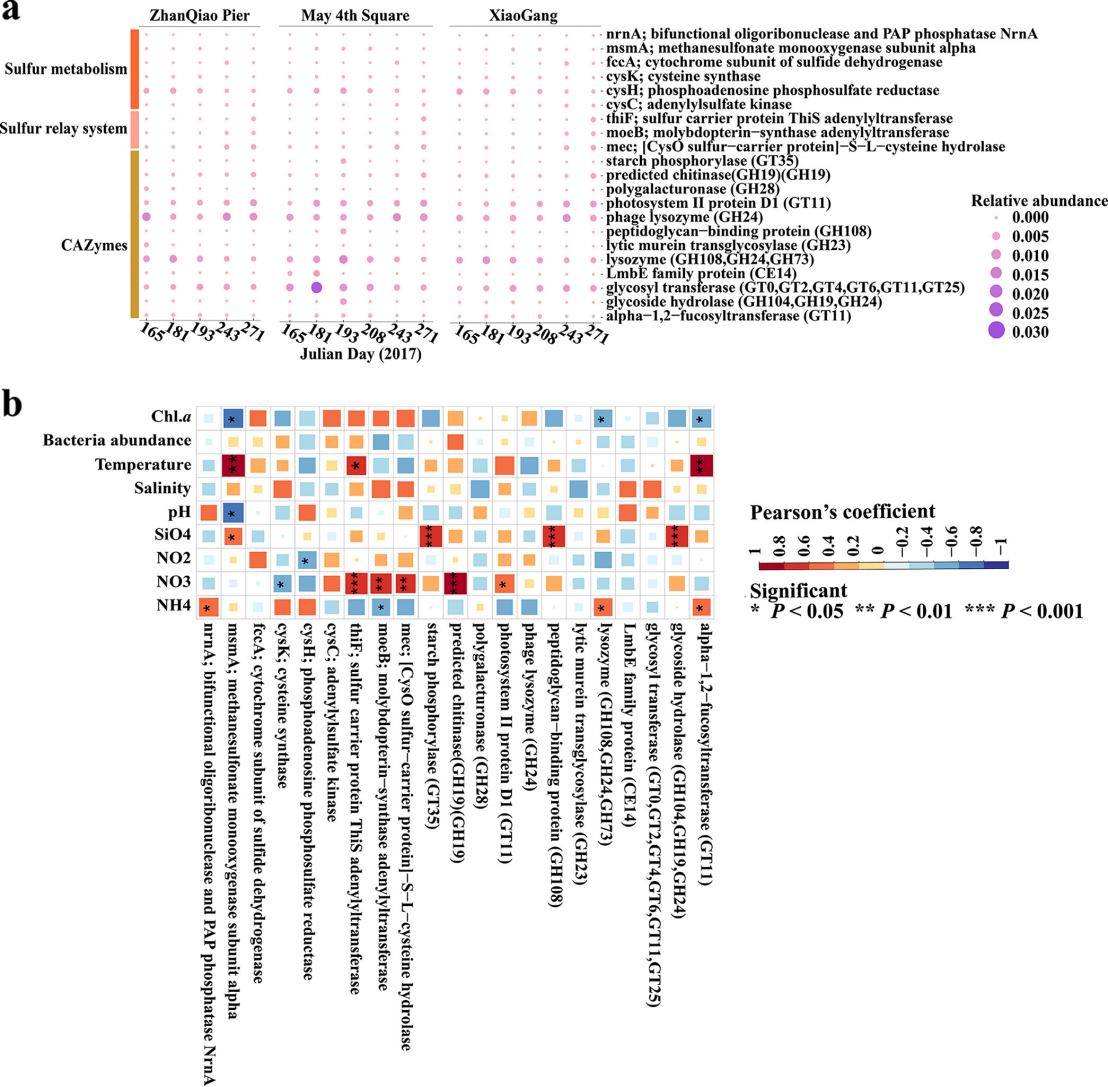
The functional gene changes caused by the arrival of U. prolifera. (a) Viral auxiliary metabolic genes (AMGs) changed during the different stages of U. prolifera bloom in the three stations. The size and shade of each circle are proportional to the relative abundance of the gene. (b) Relationship between AMGs and environmental factors. The size and shade of each square are proportional to degree of correlation. Blue and red represent positive and negative correlations between genes and environmental factors, respectively. Chl.a, chlorophyll a.

Overall, QDCV data sets were found to encode AMGs for carbohydrate, amino acid, cofactor/vitamin, and energy metabolism based on VIBRANT annotations (Fig. S5a) (27). Six and three AMGs were affiliated with sulfur metabolism and the sulfur relay system, respectively (Fig. 6a). The AMGs relating to the sulfur relay system, such as the *thiF*, *moeB*, and *mec*, were more abundant after the bloom, whereas the AMGs relating to sulfur metabolism, such as *cysK*, *cysH*, and *msmA*, were more abundant during the bloom (Fig. 6a). CAZyme genes, such as the “lysozyme,” “α-1,2-fucosyltransferase,” “glycosyl transferase,” and “glycoside hydrolase” families, were abundant during the bloom, whereas “phage lysozyme,” “photosystem II protein D1,” and “predicted chitinase” were abundant after the bloom (Fig. 6a). In addition, the “polygalacturonase,” “peptideoglycan-binding protein,” “lytic murein transglycosylase,” and LmbE family proteins were detected during the bloom. Pearson correlation analysis showed that several dominant CAZyme genes and genes related to sulfur metabolism and sulfur relay system were correlated with temperature, pH, NO_2_, NO_3_, NH_4_, SiO_3_, and chl. *a* (*P* < 0.05) (Fig. 6b).

10.1128/msystems.01211-22.5 FIG S5 (a) Viral operational taxonomic units (vOTUs) encoding carbohydrate-active enzymes changed during different stages of U. prolifera bloom in the three stations. (b) KEGG function category of viral auxiliary metabolic genes (AMGs). (c) The top 20 protein families changed during different stages of U. prolifera bloom in the three stations. Download FIG S5, TIF file, 0.9 MB. Copyright © 2023 Han et al. 2023 Han et al. https://creativecommons.org/licenses/by/4.0/ This content is distributed under the terms of the Creative Commons Attribution 4.0 International license.

## DISCUSSION

Although the world’s largest macroalgal green tide, caused by U. prolifera in the Yellow Sea, has resulted in serious social and environmental consequences and has had serious impacts on the coastal microbial community structure and metabolic activity (9, 10, 28), the impacts and responses of coastal viromes is still unknown. This study has shown that green tides significantly influence the spatiotemporal dynamics of the viral community structure, diversity, and potential function and that viruses may play potentially important roles in the biodegradation of the very large amount of organic matter released by the massive macroalgal green tide.

### Spatiotemporal patterns of the known and unknown viruses in the QDCV and a coupled dynamic with their putative hosts.

After comparing the QDCV with the NCBI Viral RefSeq and IMG/VR v3.0 (29), it was found to contain a large fraction of unknown viruses (Fig. 2 to 4; Fig. S2 and S3), suggesting that marine viruses in general are still largely uncharacterized (30–34). In general, the spatiotemporal dynamics of abundance, community structure, and diversity of the viral assemblages (Fig. 1e, 2, and 4; Fig. S1 and S3) were coupled with those of their putative hosts (Fig. 4 and 5; Fig. S4), along with the accumulation of massive amounts of algal-derived DOM after the arrival of U. prolifera (9, 10, 28). Massive algal-derived labile DOM promoted the rapid growth of bacteria and might have driven a rise in the corresponding abundance of viruses (Fig. 1e). By day 208, most of the labile DOM may have been consumed by bacteria (9, 10), and the bacterial and viral abundances gradually decreased (Fig. 1e). After the bloom, the bacterial-derived DOM (including the remnants of dead bacteria and bacterial secretions) made an important contribution to the DOM pool (9, 10) and may have driven an increase in viral diversity (Fig. S1d) and dominant vOTUs (Fig. 2b).

Due to a large proportion of unknown viruses, dynamic patterns of all vOTUs at the different stages of the bloom were characterized. Both the known (taxonomically classified viruses) and unknown (including the unclassified and previously unknown viruses) viruses had either a single or a wide peak of abundance on one or successive Julian days. Only 7% to 11% vOTUs had several peaks (Fig. 2b and c). This suggests that most viruses were influenced by the U. prolifera bloom, although there were a few that may have had a relatively stable abundance. Abundant viruses with a peak after the bloom (single 3) or during the bloom (single 1, wide 1, and wide 2), together with the CCA results (Fig. 2a), may indicate differences in the viral communities between during and after the bloom.

Based on the results of viral annotations and host prediction, it is speculated that the dynamics of these known viruses, although only a minority, may also be closely related to their hosts at different stages of the bloom. The most abundant known Pelagibacter phages were more abundant after the bloom (Fig. 4b), which show a similar temporal pattern to that of their hosts, Candidatus Pelagibacter (SAR11 clade) (Liu et al., submitted; Table S5). It is speculated that the eutrophic environment during the bloom was not suitable for the growth of the oligotrophic SAR11 clade (35). Some phages whose potential hosts were associated with degradation of algae-derived organic matter were found, such as the Sulfitobacter (36), Cycloclasticus (37), Vibrio (10, 38, 39), Bacilli (40), and Roseobacter (41) (Tables S3 and S4). These phages had similar temporal distribution patterns to their hosts, or there was a time delay between the peak in relative abundances of the virus OTUs and the putative host OTUs. Synechococcus phages were also abundant after the bloom (Fig. 4b), whereas their putative hosts had a peak abundance during the bloom (Liu et al., submitted; Table S5). Synechococcus are well known to grow in relatively high nutrient conditions and are abundant in coastal and estuarine waters (42–45). The covariation in abundance of Synechococcus spp. and cyanophages has been found in some estuarine ecosystems (42, 46). Eutrophic environments in this study may also promote the growth of Synechococcus, which correspondingly caused an increase in Synechococcus phages after the bloom. Verrucomicrobia has been reported to possess numerous pathways for the assimilation of cyanobacterial extracellular polymeric substances during cyanobacterial blooms (47) and has also acquired a complex machinery for the degradation of brown macroalgal polysaccharide fucoidans (48). In this study, the increased abundance of a *Verrucomicrobia* phage (Fig. 4b) and its putative hosts after blooms (Liu et al., submitted; Table S5) may be due to the polysaccharide released by cyanobacteria or U. prolifera.

10.1128/msystems.01211-22.8 TABLE S3 Putative virus-host linkages. Download Table S3, XLSX file, 0.03 MB. Copyright © 2023 Han et al. 2023 Han et al. https://creativecommons.org/licenses/by/4.0/ This content is distributed under the terms of the Creative Commons Attribution 4.0 International license.

10.1128/msystems.01211-22.9 TABLE S4 Relative abundance of viruses whose hosts were predicted. Download Table S4, XLSX file, 0.01 MB. Copyright © 2023 Han et al. 2023 Han et al. https://creativecommons.org/licenses/by/4.0/ This content is distributed under the terms of the Creative Commons Attribution 4.0 International license.

10.1128/msystems.01211-22.10 TABLE S5 Relative abundance of putative hosts. Download Table S5, XLSX file, 0.02 MB. Copyright © 2023 Han et al. 2023 Han et al. https://creativecommons.org/licenses/by/4.0/ This content is distributed under the terms of the Creative Commons Attribution 4.0 International license.

Due to limitations in the current virus databases and metagenomic analysis techniques, most vOTUs are unknown. However, their dominance in Qingdao coastal environments suggests that they may infect some abundant bacterial and eukaryotic populations that have not yet been identified. Since unknown viral populations account for a large portion of the viruses, and since their potential hosts and ecological role still remain largely unknown, it is clearly necessary to better understand these cryptic viral groups.

### Potential virus-mediated sulfur and carbon metabolism of the organic matter released by green tides.

As viruses carry and express some AMGs to mediate the metabolism of the host cells, such as genes involved in photosynthesis, carbon, sulfur and nitrogen metabolism, viruses can indirectly affect biogeochemical cycles (14). In this study, AMGs for sulfur metabolic cycles and CAZyme genes were found in the QDCV database (Fig. 6), which suggests that the viruses may assist their hosts with the degradation of the DOM from the U. prolifera blooms during the infection process and thus provide energy for their own reproduction. *cysC*, *cysK*, and *cysH* are predicted to participate in assimilatory sulfate reduction, and *msmA* is capable of degrading methanesulfonate (49); all of these AMGs have a wide geographical distribution (50). The *moeB*-related sulfur relay system genes were most prevalent in archaeal viral genomes and are involved in the ubiquitination process (51). Through post-translational modification of proteins, this system regulates several cellular processes, making it an ideal target for viruses to facilitate viral replication (52). *Mec* is a sulfur carrier protein that participates in l-cysteine biosynthesis and has not been found in other viral genomes. However, some other viral-encoded sulfur carrier protein, such as *dsrC*, *tusE*-like, and *soxYZ*, have been found in the Integrated Microbial Genomes/Viruses (IMG/VR v2.1) database, and phage sulfur carriers were found to be more abundant than catalytic subunits due to the greater need for sulfur carriers to drive dissimilatory sulfur transformations (53). These vOTU-encoding AMGs related to the sulfur metabolism and sulfur relay system were more abundant during the bloom and postbloom stages, respectively (Fig. 6a), suggesting their potential roles in the degradation of sulfated DOM (3).

Most AMGs were affiliated with carbohydrate metabolism, indicating that viruses can influence the prokaryotic metabolisms of carbon cycling and organic carbon decomposition during the different stages of the bloom (Fig. 6a). The high abundance of vOTUs encoding glycosyltransferase families (including α-1,2-fucosytransferase and other unclassified GT families) and glycoside hydrolase families (including polygalacturonase, lytic murein transglycosylase, and other unclassified GH families) during the bloom stage might indicate that the viruses encoding AMGs can assist bacteria to degrade the large amount DOC from U. prolifera (Fig. 6a) (3, 9); this is supported by the presence of AMGs related to central carbon metabolism and viral replication in other marine environments (54). Virus-encoded CAZyme genes have also been proposed to enhance the breakdown of complex carbohydrates to promote the metabolism and energy production of the host during viral infection (55, 56). In this study, several novel AMGs related to carbohydrate metabolism were detected, such as α-1,2-fucosytransferase, polygalacturonase, and lytic murein transglycosylase (Fig. 6a).

Massive macroalgal blooms can cause the death of other chitin-rich organisms, including protozoa and invertebrates, through the development of hypoxia (3, 9). In this study, the relative abundance of vOTUs encoding the chitinase and peptidoglycan-binding protein increased at the TB stage or after bloom (Fig. 6a), suggesting that the viruses may be related to chitin-degradation bacteria (57). Viral-encoded chitinase has been detected in flavobacterial phages, whose hosts are usually abundant during algal bloom and may degrade the bacterial peptidoglycan function (58, 59).

### Conclusion.

This study, for the first time, used a metagenomic analysis to investigate the community structure, diversity, life strategy, and AMGs of DNA viruses in coastal waters during a U. prolifera bloom. A large proportion of the viruses identified were novel, and the viral community structure (both of the known and unknown viruses) and AMGs showed a clear succession during the different stages of the bloom, which was consistent with the spatiotemporal dynamics of their putative hosts. For the known viruses, viruses linked to hosts with degradation of algal-derived organic matter were found, such as Sulfitobacter, Cycloclasticus, Vibrio, Bacilli, and Roseobacter phages. AMGs related to sulfur (such as *cysC*, *cysK*, *cysH*, and *msmA*) and carbon (such as polygalacturonase, α-1,2-fucosytransferase, and chitinase) metabolism were also discovered, and they may assist the hosts to degrade the algal-derived and other DOM. Whether the viruses will assist the host in degrading the liable, semiliable, and refractory DOM requires further study. This study has enhanced our understanding of the interactions between viruses and their hosts and created a preliminary view of the ecological roles of viruses in coastal waters affected by the largest U. prolifera green tide in the world.

## MATERIALS AND METHODS

### Sample collection and detection of environmental factors.

Seventeen seawater samples (~20 liters) were collected from 5 m beneath the surface at three stations in Qingdao City: ZQ (36°3′43.2″N,120°18′54″E), MS (36°3′43.2″N, 120°22′48″E), and XG (36°4′37.2″N, 120°18′32.4″E) along the coastal waters of Yellow Sea from 14 June to 28 September in 2017 (Fig. 1a). This included six time points (Julian days 165, 181, 193, 208, and 271) and covered the bloom stages from the arrival of the bloom to 2 months after the bloom in 2017. All of the seawater samples were filtered through 200-, 3.0-, and 0.2-μm filters (Millipore, Burlington, MA, USA) to remove particles and larger cellular microorganisms. The 3.0- and 0.2-μm filters were used for 18S and 16S sequencing analysis, respectively (Liu et al., submitted). Seawater was collected with Niskin bottles for nutrient (SiO_3_, NH_4_, NO_3_, and NO_2_) and chl. *a* concentration and were determined by a four-channel AA3 continuous flow AutoAnalyzer (60) and the extractive fluorescence method on a Turner Designs 10-AU field fluorometer (61), respectively. Triplicate samples (1.5 mL) for viral and bacterial abundance were fixed with glutaraldehyde (final concentration, 0.5%), frozen in liquid nitrogen, and stored at −80°C until analysis (62).

### Viral and bacterial abundance.

Viral abundance was estimated following the previous methods (62, 63) with some modifications. The fixed and frozen samples (−80°C) were thawed at 37°C, diluted 10- to 100-fold with 0.02-μm filtered Tris-EDTA buffer (pH 8) (Sigma-Aldrich, Hamburg, Germany) and stained with SYBR green I (final concentration of 0.5 × 10^−4^ of the Molecular Probes stock solution, Thermo Fisher, USA) in the dark at 80°C. Then, the incubated samples were cooled at room temperature for 5 min and analyzed with a CytoFLEX flow cytometer (Beckman, Shanghai, China) at a total volume of 30 μL sample^−1^. For bacterial enumeration (63, 64), the thawed samples were diluted 10-fold with 0.02-μm filtered Tris-EDTA buffer, stained with SYBR Gold (final dilution of 10^−4^ of the commercial stock solution) for 15 min in the dark, and analyzed with the CytoFLEX flow cytometer (Beckman) for 1 min at a delivery rate of 60 μL min^−1^.

### Viral concentration, DNA extraction, and metagenomic sequencing.

The FeCl_3_-mediated flocculation method (65) was used to enrich the viruses. In brief, 2.5 mL of 10 g/liter Fe stock (FeCl_3_) solution was added to the filtered seawater with a 0.2-μm filter and mixed thoroughly. After incubating at room temperature for at least 40 min, the sample was passed through a polycarbonate membrane filter (0.8 μm; Millipore) and stored at 4°C until analysis.

The filtered membranes with concentrated viral particles were resuspended in 0.1 M EDTA, 0.2 M MgCl_2_ buffer (pH 6.0) at 4°C. The viruses were concentrated to about 400 μL by centrifugal ultrafiltration (membrane package: 122 Pellicon 2 Cassette, Biomax, 50 kDa; polyethersulfone). Total DNA was extracted using the QIAamp DNA minikit (Qiagen) according to the manufacturer’s instructions. Library construction was conducted using a NEBNext Ultra DNA Library Prep kit (New England Biolabs, Ipswich, MA, USA), and high-throughput sequencing of the viral DNA was performed using the Illumina NovaSeq 6000 (pair-end sequencing, 2 × 150 bp) platform (Novogene Bioinformatics Technology Co., Ltd., Nanjing, China).

### Quality control, assembly, and identification of viral contigs.

The raw reads were removed from the adapters using Cutadapt (66), and the high-quality and paired-end reads were filtered by Perl scripts using the following conditions: (i) no more than 20% of bases with a quality score less than 20 and (ii) no more than 30% of bases with a quality score less than 30 (67). High-quality reads in each sample were assembled using metaSPAdes (v3.12.0) (68). Contigs shorter than 3 kb were removed. The quality of these metagenomic assemblies was evaluated using MetaQUAST (v5.0.2) (69). Then, VirFinder and VirSorter (v1.0.6) were used to identify the viral contigs (70, 71). Identification criteria for viral contigs: (i) VirFinder with a score ≥ 0.9 and *P *< 0.05; (ii) VirSorter in categories 1, 2, 4, and 5; (iii) VirFinder with a score ≥ 0.7 and *P *< 0.05 and VirSorter in categories 1 to 6; and (iv) contigs detected as virus by CAT, which utilizes the last common ancestor (LCA) of conservative open reading frames (ORFs) to determine the taxon (72).

### Dereplication, calculating relative abundances, and taxonomic profiling.

The identified viral contigs from each sample were clustered at 95% nucleotide identity across ≥80% of shorter viral contigs using CD-HIT v4.8.1 (parameters: -c 0.95 -G 0 -M 0 -aS 0.8 -T 8 -n 9), producing 28,058 viral operational taxonomic units (vOTUs). To calculate the relative abundances of vOTUs in each sample, clean reads were mapped to the vOTUs using bowtie2 (73) and counted using CoverM v0.3.1 (https://github.com/wwood/CoverM) (parameters: contig mode for vOTUs, -min-read-percent-identity 0.95 -min-read-aligned-percent 0.75). Then, the relative abundances of the vOTUs were determined as transcripts per million (TPM) reads mapped (74).

The viral taxonomic classifications of the vOTUs were annotated by CAT (72); the ORFs were predicted using Prodigal and then searched against the NCBI-nr database (version of 18 June 2020), and the viral taxonomic classifications were generated based on the LCA scores of ORFs. The NCBI database was manually searched against to correct the taxonomic information when necessary. The lifestyle of the viruses (including the lytic and lysogenic lifestyle) was determined by VIBRANT (v1.2.1) using the default parameters (27).

### Virus-host prediction.

The virus-host linkages were predicted using three different *in silico* methods, including CRISPR spacer match, tRNA match, and nucleotide sequence homology (75): (i) Sequences of vOTUs (≥5 kb) were compared with bacterial genomes (bacterial NCBI RefSeq, version of 31 July 2021) using BLASTn (query coverage ≥ 75%; identity ≥ 70%; bit score ≥ 50; and E value ≤ 1 × 10^−5^). (ii) The tRNAs from sequences of vOTUs and bacterial genomes were identified using ARAGORN (v1.2.38) (parameters: -t) (76). Each match by reciprocal BLASTN required ≥90% length with identity ≥90% of the sequences (77). (iii) CRISPR arrays were predicted from bacterial genomes by metaCRT (78), and CRISPR spacers were compared with the viral contigs using BLASTN with an E value threshold of 1.0 × 10^−5^ and ≤1 mismatch over the whole spacer (79).

### Functional annotation and abundance of ORFs and AMGs.

ORFs were predicted using metaProdigal (80). VIBRANT annotations were performed on viral contigs, and the categories “metabolic pathways” and “sulfur relay system” were considered potential AMGs (27). To further identify the protein domains, Pfamscan was applied by comparing viral ORFs to the PfamScan database (E value < 1 × 10^−5^; bit score > 30; version Pfam33.1) (81). To detect the AMGs of viral contigs, the viral ORFs were blastp against the CAZymes database (version of 30 July 2020) to find glycol metabolism-related genes using DIAMOND (e value < 1 × 10^−5^; bit score > 50) and searched for sulfur and nitrogen metabolic genes using GhostKOALA against the KEGG GENES database (https://www.kegg.jp/ghostkoala/, version 2.2). The relative abundance of viral ORFs was calculated for each sample by summing the relative abundance of each ORF and normalizing to the abundance of the vOTUs in which it was encoded.

### Co-occurrence network analysis.

To cluster and place the vOTUs (≥10 kb) in the context of known viruses, the predicted proteins were clustered with predicted proteins from viral sequences in public databases (v201, version of 31 July 2021). All proteins were compared using all-versus-all DIAMOND (v0.9.29.130) BLASTp (E value≤ 1 × 10^−5^; query coverage ≥ 50%; identity ≥ 25%) (82). A similarity score for each pair was calculated as the negative logarithmic score by multiplying the hypergeometric similarity *P* value by the total number of pairwise comparison using vConTACT2 (https://bitbucket.org/MAVERICLab/vcontact2, accessed 8 May 2020). For comparison with other environmental viromes, we also placed the vOTUs (≥ 10kb) with the viral reference (4,086 vOTUs) and high-quality (21,148 vOTUs) predicted complete viral genomes (vOTUs) from marine, freshwater, terrestrial soil, wastewater environment, and algae-host-associated genomes in IMG/VR v3 data set (29) using vConTACT2.

The network structure was used to determine the potential interactions between viral and host communities based on the Pearson index between viral clusters (VCs) and host OTUs. Only robust (*r *> 0.8 or *r* < −0.8) and statistically significant (*P *< 0.05) correlations were included into the network analysis.

### Statistical analysis.

All statistical analyses were performed in R. α-Diversity and β-diversity of viral communities were calculated using vegan package v2.5. PCA and CCA based on Bray-Curtis dissimilarities were generated from vOTU tables with viral abundances (TPM) using the vegdist function (method “bray”). For analysis of vOTUs and VCs dynamics, higher than the mean value of abundance (TPM) for a vOTU/VC was considered belonging to a peak of abundance (83). The vOTUs/VCs had three kinds of peaks in relative abundance: (i) single peak (including vOTU/VC peaks of relative abundance on days 165 and 181, single 1; on days 193 and 208, single 2; on days 243 and 271, single 3; and on days 165 and 271, single 4), (ii) wide peaks, which means the peak occurs in 1 or 2 adjacent Julian days on the basis of single types (again, including all four subtypes), and (iii) several peaks (except to single and wide peaks types) of abundance.

### Availability of data.

The raw reads data reported in this paper were previously deposited in the NCBI database (under Bioproject number PRJNA797266).

## References

[1] Smetacek V, Zingone A. 2013. Green and golden seaweed tides on the rise. Nature 504:84–88. doi:10.1038/nature12860. 24305152

[2] Xiao J, Wang Z, Liu D, Fu M, Yuan C, Yan T. 2021. Harmful macroalgal blooms (HMBs) in China’s coastal water: green and golden tides. Harmful Algae 107:102061. doi:10.1016/j.hal.2021.102061. 34456020

[3] Zhang Y, He P, Li H, Li G, Liu J, Jiao F, Zhang J, Huo Y, Shi X, Su R, Ye N, Liu D, Yu R, Wang Z, Zhou M, Jiao N. 2019. *Ulva prolifera* green-tide outbreaks and their environmental impact in the Yellow Sea, China. Natl Sci Rev 6:825–838. doi:10.1093/nsr/nwz026. 34691936 PMC8291432

[4] Zhang T, Wang X. 2017. Release and microbial degradation of dissolved organic matter (DOM) from the macroalgae *Ulva prolifera*. Mar Pollut Bull 125:192–198. doi:10.1016/j.marpolbul.2017.08.029. 28821354

[5] Nemergut DR, Schmidt SK, Fukami T, O’Neill SP, Bilinski TM, Stanish LF, Knelman JE, Darcy JL, Lynch RC, Wickey P, Ferrenberg S. 2013. Patterns and processes of microbial community assembly. Microbiol Mol Biol Rev 77:342–356. doi:10.1128/MMBR.00051-12. 24006468 PMC3811611

[6] Li H, Zhang Y, Han X, Shi X, Rivkin RB, Legendre L. 2016. Growth responses of *Ulva prolifera* to inorganic and organic nutrients: implications for macroalgal blooms in the southern Yellow Sea, China. Sci Rep 6:26498. doi:10.1038/srep26498. 27199215 PMC4873802

[7] Zhang X, Song Y, Liu D, Keesing JK, Gong J. 2015. Macroalgal blooms favor heterotrophic diazotrophic bacteria in nitrogen-rich and phosphorus-limited coastal surface waters in the Yellow Sea. Estuar Coast Shelf Sci 163:75–81. doi:10.1016/j.ecss.2014.12.015.

[8] Marshall K, Joint I, Callow ME, Callow JA. 2006. Effect of marine bacterial isolates on the growth and morphology of axenic plantlets of the green alga *Ulva linza*. Microb Ecol 52:302–310. doi:10.1007/s00248-006-9060-x. 16897307

[9] Chen J, Li H, Zhang Z, He C, Shi Q, Jiao N, Zhang Y. 2020. DOC dynamics and bacterial community succession during long-term degradation of *Ulva prolifera* and their implications for the legacy effect of green tides on refractory DOC pool in seawater. Water Res 185:116268. doi:10.1016/j.watres.2020.116268. 32784034

[10] Liang J, Liu J, Zhan Y, Zhou S, Xue C-X, Sun C, Lin Y, Luo C, Wang X, Zhang X-H. 2021. Succession of marine bacteria in response to *Ulva prolifera*-derived dissolved organic matter. Environ Int 155:106687. doi:10.1016/j.envint.2021.106687. 34144477

[11] Fuhrman JA. 1999. Marine viruses and their biogeochemical and ecological effects. Nature 399:541–548. doi:10.1038/21119. 10376593

[12] Suttle CA. 2007. Marine viruses–major players in the global ecosystem. Nat Rev Microbiol 5:801–812. doi:10.1038/nrmicro1750. 17853907

[13] Breitbart M. 2012. Marine viruses: truth or dare. Annu Rev Mar Sci 4:425–448. doi:10.1146/annurev-marine-120709-142805. 22457982

[14] Zimmerman AE, Howard-Varona C, Needham DM, John SG, Worden AZ, Sullivan MB, Waldbauer JR, Coleman ML. 2020. Metabolic and biogeochemical consequences of viral infection in aquatic ecosystems. Nat Rev Microbiol 18:21–34. doi:10.1038/s41579-019-0270-x. 31690825

[15] Alarcon-Schumacher T, Guajardo-Leiva S, Anton J, Diez B. 2019. Elucidating viral communities during a phytoplankton bloom on the West Antarctic Peninsula. Front Microbiol 10:1014. doi:10.3389/fmicb.2019.01014. 31139164 PMC6527751

[16] Kranzler CF, Krause JW, Brzezinski MA, Edwards BR, Biggs WP, Maniscalco M, McCrow JP, Van Mooy BAS, Bidle KD, Allen AE, Thamatrakoln K. 2019. Silicon limitation facilitates virus infection and mortality of marine diatoms. Nat Microbiol 4:1790–1797. doi:10.1038/s41564-019-0502-x. 31308524

[17] Martinez JM, Schroeder DC, Wilson WH. 2012. Dynamics and genotypic composition of *Emiliania huxleyi* and their co-occurring viruses during a coccolithophore bloom in the North Sea. FEMS Microbiol Ecol 81:315–323. doi:10.1111/j.1574-6941.2012.01349.x. 22404582

[18] Morimoto D, Sulcius S, Yoshida T. 2020. Viruses of freshwater bloom-forming cyanobacteria: genomic features, infection strategies and coexistence with the host. Environ Microbiol Rep 12:486–502. doi:10.1111/1758-2229.12872. 32754956

[19] Malitsky S, Ziv C, Rosenwasser S, Zheng SN, Schatz D, Porat Z, Ben-Dor S, Aharoni A, Vardi A. 2016. Viral infection of the marine alga *Emiliania huxleyi* triggers lipidome remodeling and induces the production of highly saturated triacylglycerol. New Phytol 210:88–96. doi:10.1111/nph.13852. 26856244

[20] Schleyer G, Shahaf N, Ziv C, Dong YH, Meoded RA, Helfrich EJN, Schatz D, Rosenwasser S, Rogachev I, Aharoni A, Piel J, Vardi A. 2019. In plaque-mass spectrometry imaging of a bloom-forming alga during viral infection reveals a metabolic shift towards odd-chain fatty acid lipids. Nat Microbiol 4:527–538. doi:10.1038/s41564-018-0336-y. 30718847 PMC6420086

[21] Vardi A, Van Mooy BAS, Fredricks HF, Popendorf KJ, Ossolinski JE, Haramaty L, Bidle KD. 2009. Viral glycosphingolipids induce lytic infection and cell death in marine phytoplankton. Science 326:861–865. doi:10.1126/science.1177322. 19892986

[22] Sabbagh EI, Huete-Stauffer TM, Calleja MLL, Silva L, Viegas M, Moran XAG. 2020. Weekly variations of viruses and heterotrophic nanoflagellates and their potential impact on bacterioplankton in shallow waters of the central Red Sea. FEMS Microbiol Ecol 96:fiaa033. doi:10.1093/femsec/fiaa033. 32149360 PMC7104677

[23] Laber CP, Hunter JE, Carvalho F, Collins JR, Hunter EJ, Schieler BM, Boss E, More K, Frada M, Thamatrakoln K, Brown CM, Haramaty L, Ossolinski J, Fredricks H, Nissimov JI, Vandzura R, Sheyn U, Lehahn Y, Chant RJ, Martins AM, Coolen MJL, Vardi A, DiTullio GR, Van Mooy BAS, Bidle KD. 2018. Coccolithovirus facilitation of carbon export in the North Atlantic. Nat Microbiol 3:537–547. doi:10.1038/s41564-018-0128-4. 29531367

[24] Kuhlisch C, Schleyer G, Shahaf N, Vincent F, Schatz D, Vardi A. 2021. Viral infection of algal blooms leaves a unique metabolic footprint on the dissolved organic matter in the ocean. Sci Adv 7:eabf4680. doi:10.1126/sciadv.abf4680. 34144983 PMC8213229

[25] Wang C, Yu R-C, Zhou M-J. 2012. Effects of the decomposing green macroalga *Ulva* (*Enteromorpha*) *prolifera* on the growth of four red-tide species. Harmful Algae 16:12–19. doi:10.1016/j.hal.2011.12.007.

[26] Zhao JY, Geng HX, Zhang QC, Li YF, Kong FZ, Yan T, Zhou MJ, Yang D, Yuan Y, Yu RC. 2022. Green tides in the Yellow Sea promoted the proliferation of pelagophyte *Aureococcus anophagefferens*. Environ Sci Technol 56:3056–3064. doi:10.1021/acs.est.1c06502. 35133807

[27] Kieft K, Zhou Z, Anantharaman K. 2020. VIBRANT: automated recovery, annotation and curation of microbial viruses, and evaluation of viral community function from genomic sequences. Microbiome 8:90. doi:10.1186/s40168-020-00867-0. 32522236 PMC7288430

[28] Wang J, Lu J, Zhang Y, Wu J. 2020. Microbial ecology might serve as new indicator for the influence of green tide on the coastal water quality: assessment the bioturbation of *Ulva prolifera* outbreak on bacterial community in coastal waters. Ecol Indicators 113:106211. doi:10.1016/j.ecolind.2020.106211.

[29] Roux S, Paez Espino D, Chen IM, Palaniappan K, Ratner A, Chu K, Reddy T, Nayfach S, Schulz F, Call L, Neches R, Woyke T, Ivanova N, Eloe-Fadrosh E, Kyrpides N. 2021. IMG/VR v3: an integrated ecological and evolutionary framework for interrogating genomes of uncultivated viruses. Nucleic Acids Res 49:D764–D775. doi:10.1093/nar/gkaa946. 33137183 PMC7778971

[30] Gao C, Xia J, Zhou X, Liang Y, Jiang Y, Wang M, Shao H, Shi X, Guo C, He H, Wang H, He J, Hu D, Wang X, Zhao J, Zhang YZ, Sung YY, Mok WJ, Wong LL, McMinn A, Suttle CA, Wang M. 2021. Viral characteristics of the warm Atlantic and cold Arctic water masses in the Nordic Seas. Appl Environ Microbiol 87:e0116021. doi:10.1128/AEM.01160-21. 34469192 PMC8552889

[31] Gu C, Liang Y, Li J, Shao H, Jiang Y, Zhou X, Gao C, Li X, Zhang W, Guo C, He H, Wang H, Sung YY, Mok WJ, Wong LL, Suttle CA, McMinn A, Tian J, Wang M. 2021. Saline lakes on the Qinghai-Tibet Plateau harbor unique viral assemblages mediating microbial environmental adaption. iScience 24:103439. doi:10.1016/j.isci.2021.103439. 34988389 PMC8710556

[32] Liang Y, Wang L, Wang Z, Zhao J, Yang Q, Wang M, Yang K, Zhang L, Jiao N, Zhang Y. 2019. Metagenomic analysis of the diversity of DNA viruses in the surface and deep sea of the South China Sea. Front Microbiol 10:1951. doi:10.3389/fmicb.2019.01951. 31507563 PMC6716333

[33] Gregory AC, Zayed AA, Conceicao-Neto N, Temperton B, Bolduc B, Alberti A, Ardyna M, Arkhipova K, Carmichael M, Cruaud C, Dimier C, Dominguez-Huerta G, Ferland J, Kandels S, Liu Y, Marec C, Pesant S, Picheral M, Pisarev S, Poulain J, Tremblay JE, Vik D, Tara Oceans C, Babin M, Bowler C, Culley AI, de Vargas C, Dutilh BE, Iudicone D, Karp-Boss L, Roux S, Sunagawa S, Wincker P, Sullivan MB, Tara Oceans Coordinators. 2019. Marine DNA viral macro- and microdiversity from pole to pole. Cell 177:1109–1123.e14. doi:10.1016/j.cell.2019.03.040. 31031001 PMC6525058

[34] Gong Z, Liang Y, Wang M, Jiang Y, Yang Q, Xia J, Zhou X, You S, Gao C, Wang J, He J, Shao H, McMinn A. 2018. Viral diversity and its relationship with environmental factors at the surface and deep sea of Prydz Bay, Antarctica. Front Microbiol 9:2981. doi:10.3389/fmicb.2018.02981. 30559737 PMC6287040

[35] Carini P, Steindler L, Beszteri S, Giovannoni SJ. 2013. Nutrient requirements for growth of the extreme oligotroph “*Candidatus Pelagibacter ubique*” HTCC1062 on a defined medium. ISME J 7:592–602. doi:10.1038/ismej.2012.122. 23096402 PMC3578571

[36] Gnaim R, Polikovsky M, Unis R, Sheviryov J, Gozin M, Golberg A. 2021. Marine bacteria associated with the green seaweed *Ulva* sp. for the production of polyhydroxyalkanoates. Bioresour Technol 328:124815. doi:10.1016/j.biortech.2021.124815. 33609888

[37] Wang W, Wang L, Shao Z. 2018. Polycyclic aromatic hydrocarbon (PAH) degradation pathways of the obligate marine PAH degrader *Cycloclasticus* sp. strain P1. Appl Environ Microbiol 84:e01261-18. doi:10.1128/AEM.01261-18. 30171002 PMC6193391

[38] Zhang X, Lin H, Wang X, Austin B. 2018. Significance of *Vibrio* species in the marine organic carbon cycle—a review. Sci China Earth Sci 61:1357–1368. doi:10.1007/s11430-017-9229-x.

[39] Westrich JR, Ebling AM, Landing WM, Joyner JL, Kemp KM, Griffin DW, Lipp EK. 2016. Saharan dust nutrients promote *Vibrio* bloom formation in marine surface waters. Proc Natl Acad Sci USA 113:5964–5969. doi:10.1073/pnas.1518080113. 27162369 PMC4889353

[40] Guan C, Guo X, Cai G, Zhang H, Li Y, Zheng W, Zheng T. 2014. Novel algicidal evidence of a bacterium *Bacillus* sp. LP-10 killing *Phaeocystis globosa*, a harmful algal bloom causing species. Biol Control 76:79–86. doi:10.1016/j.biocontrol.2014.05.007.

[41] Miller TR, Belas R. 2004. Dimethylsulfoniopropionate metabolism by *Pfiesteria*-associated *Roseobacter* spp. Appl Environ Microbiol 70:3383–3391. doi:10.1128/AEM.70.6.3383-3391.2004. 15184135 PMC427730

[42] Wang K, Wommack KE, Chen F. 2011. Abundance and distribution of *Synechococcus* spp. and cyanophages in the Chesapeake Bay. Appl Environ Microbiol 77:7459–7468. doi:10.1128/AEM.00267-11. 21821760 PMC3209163

[43] Affronti LF, Jr, Marshall HG. 1993. Diel abundance and productivity patterns of autotrophic picoplankton in the lower Chesapeake Bay. J Plankton Res 15:1–8. doi:10.1093/plankt/15.1.1.

[44] Murrell MC, Lores EM. 2004. Phytoplankton and zooplankton seasonal dynamics in a subtropical estuary: importance of cyanobacteria. J Plankton Res 26:371–382. doi:10.1093/plankt/fbh038.

[45] Agawin NS, Duarte C, Agustí S. 2000. Response of Mediterranean *Synechococcus* growth and loss rates to experimental nutrient inputs. Mar Ecol Prog Ser 206:97–106. doi:10.3354/meps206097.

[46] Waterbury JB, Valois FW. 1993. Resistance to co-occurring phages enables marine *Synechococcus* communities to coexist with cyanophages abundant in seawater. Appl Environ Microbiol 59:3393–3399. doi:10.1128/aem.59.10.3393-3399.1993. 16349072 PMC182464

[47] Woodhouse JN, Kinsela AS, Collins RN, Bowling LC, Honeyman GL, Holliday JK, Neilan BA. 2016. Microbial communities reflect temporal changes in cyanobacterial composition in a shallow ephemeral freshwater lake. ISME J 10:1337–1351. doi:10.1038/ismej.2015.218. 26636552 PMC5029192

[48] Sichert A, Corzett CH, Schechter MS, Unfried F, Markert S, Becher D, Fernandez-Guerra A, Liebeke M, Schweder T, Polz MF, Hehemann J-H. 2020. *Verrucomicrobia* use hundreds of enzymes to digest the algal polysaccharide fucoidan. Nat Microbiol 5:1026–1039. doi:10.1038/s41564-020-0720-2. 32451471

[49] Henriques AC, De Marco P. 2015. Methanesulfonate (MSA) catabolic genes from marine and estuarine bacteria. PLoS One 10:e0125735. doi:10.1371/journal.pone.0125735. 25978049 PMC4433239

[50] Kieft K, Breister AM, Huss P, Linz AM, Zanetakos E, Zhou Z, Rahlff J, Esser SP, Probst AJ, Raman S, Roux S, Anantharaman K. 2021. Virus-associated organosulfur metabolism in human and environmental systems. Cell Rep 36:109471. doi:10.1016/j.celrep.2021.109471. 34348151

[51] Coutinho FH, Zaragoza-Solas A, Lopez-Perez M, Barylski J, Zielezinski A, Dutilh BE, Edwards R, Rodriguez-Valera F. 2021. RaFAH: host prediction for viruses of Bacteria and Archaea based on protein content. Patterns 2:100274. doi:10.1016/j.patter.2021.100274. 34286299 PMC8276007

[52] Randow F, Lehner PJ. 2009. Viral avoidance and exploitation of the ubiquitin system. Nat Cell Biol 11:527–534. doi:10.1038/ncb0509-527. 19404332

[53] Kieft K, Zhou Z, Anderson RE, Buchan A, Campbell BJ, Hallam SJ, Hess M, Sullivan MB, Walsh DA, Roux S, Anantharaman K. 2021. Ecology of inorganic sulfur auxiliary metabolism in widespread bacteriophages. Nat Commun 12:3503. doi:10.1038/s41467-021-23698-5. 34108477 PMC8190135

[54] Hurwitz BL, U’Ren JM. 2016. Viral metabolic reprogramming in marine ecosystems. Curr Opin Microbiol 31:161–168. doi:10.1016/j.mib.2016.04.002. 27088500

[55] Anderson CL, Sullivan MB, Fernando SC. 2017. Dietary energy drives the dynamic response of bovine rumen viral communities. Microbiome 5:155. doi:10.1186/s40168-017-0374-3. 29179741 PMC5704599

[56] Jin M, Guo X, Zhang R, Qu W, Gao B, Zeng R. 2019. Diversities and potential biogeochemical impacts of mangrove soil viruses. Microbiome 7:58. doi:10.1186/s40168-019-0675-9. 30975205 PMC6460857

[57] Cottrell MT, Wood DN, Yu LY, Kirchman DL. 2000. Selected chitinase genes in cultured and uncultured marine bacteria in the alpha- and gamma-subclasses of the proteobacteria. Appl Environ Microbiol 66:1195–1201. doi:10.1128/AEM.66.3.1195-1201.2000. 10698791 PMC91962

[58] Souza CP, Almeida BC, Colwell RR, Rivera IN. 2011. The importance of chitin in the marine environment. Mar Biotechnol 13:823–830. doi:10.1007/s10126-011-9388-1. 21607543

[59] Bartlau N, Wichels A, Krohne G, Adriaenssens EM, Heins A, Fuchs BM, Amann R, Moraru C. 2022. Highly diverse flavobacterial phages isolated from North Sea spring blooms. ISME J 16:555–568. doi:10.1038/s41396-021-01097-4. 34475519 PMC8776804

[60] Bran & Luebbe AutoAnalyzer Applications: AutoAnalyzer method No. G-172-96 nitrate and nitrite in water and seawater. 1997. Bran & Luebbe, Inc., Buffalo Grove, IL.

[61] Mock T, Hoch N. 2005. Long-term temperature acclimation of photosynthesis in steady-state cultures of the polar diatom *Fragilariopsis cylindrus*. Photosynth Res 85:307–317. doi:10.1007/s11120-005-5668-9. 16170633

[62] Brussaard CP. 2004. Optimization of procedures for counting viruses by flow cytometry. Appl Environ Microbiol 70:1506–1513. doi:10.1128/AEM.70.3.1506-1513.2004. 15006772 PMC368280

[63] Liang Y, Bai X, Jiang Y, Wang M, He J, McMinn A. 2016. Distribution of marine viruses and their potential hosts in Prydz Bay and adjacent Southern Ocean, Antarctic. Polar Biol 39:365–378. doi:10.1007/s00300-015-1787-8.

[64] Marie D, Partensky F, Jacquet S, Vaulot D. 1997. Enumeration and cell cycle analysis of natural populations of marine picoplankton by flow cytometry using the nucleic acid stain SYBR Green I. Appl Environ Microbiol 63:186–193. doi:10.1128/aem.63.1.186-193.1997. 16535483 PMC1389098

[65] John SG, Mendez CB, Deng L, Poulos B, Kauffman AK, Kern S, Brum J, Polz MF, Boyle EA, Sullivan MB. 2011. A simple and efficient method for concentration of ocean viruses by chemical flocculation. Environ Microbiol Rep 3:195–202. doi:10.1111/j.1758-2229.2010.00208.x. 21572525 PMC3087117

[66] Martin M. 2011. Cutadapt removes adapter sequences from high-throughput sequencing reads. EMBnet J 17:10–12. doi:10.14806/ej.17.1.200.

[67] Yang Q, Gao C, Jiang Y, Wang M, Zhou X, Shao H, Gong Z, McMinn A. 2019. Metagenomic characterization of the viral community of the South Scotia Ridge. Viruses 11:95. doi:10.3390/v11020095. 30678352 PMC6410227

[68] Nurk S, Meleshko D, Korobeynikov A, Pevzner PA. 2017. metaSPAdes: a new versatile metagenomic assembler. Genome Res 27:824–834. doi:10.1101/gr.213959.116. 28298430 PMC5411777

[69] Mikheenko A, Saveliev V, Gurevich A. 2016. MetaQUAST: evaluation of metagenome assemblies. Bioinformatics 32:1088–1090. doi:10.1093/bioinformatics/btv697. 26614127

[70] Roux S, Enault F, Hurwitz BL, Sullivan MB. 2015. VirSorter: mining viral signal from microbial genomic data. PeerJ 3:e985. doi:10.7717/peerj.985. 26038737 PMC4451026

[71] Ren J, Ahlgren NA, Lu YY, Fuhrman JA, Sun F. 2017. VirFinder: a novel k-mer based tool for identifying viral sequences from assembled metagenomic data. Microbiome 5:69. doi:10.1186/s40168-017-0283-5. 28683828 PMC5501583

[72] von Meijenfeldt FAB, Arkhipova K, Cambuy DD, Coutinho FH, Dutilh BE. 2019. Robust taxonomic classification of uncharted microbial sequences and bins with CAT and BAT. Genome Biol 20:217. doi:10.1186/s13059-019-1817-x. 31640809 PMC6805573

[73] Langmead B, Salzberg SL. 2012. Fast gapped-read alignment with Bowtie 2. Nat Methods 9:357–359. doi:10.1038/nmeth.1923. 22388286 PMC3322381

[74] Li B, Ruotti V, Stewart RM, Thomson JA, Dewey CN. 2010. RNA-Seq gene expression estimation with read mapping uncertainty. Bioinformatics 26:493–500. doi:10.1093/bioinformatics/btp692. 20022975 PMC2820677

[75] Li ZX, Pan D, Wei GS, Pi WL, Zhang CW, Wang JH, Peng YY, Zhang L, Wang Y, Hubert CRJ, Dong XY. 2021. Deep sea sediments associated with cold seeps are a subsurface reservoir of viral diversity. ISME J 15:2366–2378. doi:10.1038/s41396-021-00932-y. 33649554 PMC8319345

[76] Laslett D, Canback B. 2004. ARAGORN, a program to detect tRNA genes and tmRNA genes in nucleotide sequences. Nucleic Acids Res 32:11–16. doi:10.1093/nar/gkh152. 14704338 PMC373265

[77] Coutinho FH, Silveira CB, Gregoracci GB, Thompson CC, Edwards RA, Brussaard CPD, Dutilh BE, Thompson FL. 2017. Marine viruses discovered via metagenomics shed light on viral strategies throughout the oceans. Nat Commun 8:15955. doi:10.1038/ncomms15955. 28677677 PMC5504273

[78] Rho M, Wu YW, Tang HX, Doak TG, Ye YZ. 2012. Diverse CRISPRs evolving in human microbiomes. PLoS Genet 8:e1002441. doi:10.1371/journal.pgen.1002441. 22719260 PMC3374615

[79] Emerson JB, Roux S, Brum JR, Bolduc B, Woodcroft BJ, Jang HB, Singleton CM, Solden LM, Naas AE, Boyd JA, Hodgkins SB, Wilson RM, Trubl G, Li C, Frolking S, Pope PB, Wrighton KC, Crill PM, Chanton JP, Saleska SR, Tyson GW, Rich VI, Sullivan MB. 2018. Host-linked soil viral ecology along a permafrost thaw gradient. Nat Microbiol 3:870–880. doi:10.1038/s41564-018-0190-y. 30013236 PMC6786970

[80] Hyatt D, Chen GL, LoCascio PF, Land ML, Larimer FW, Hauser LJ. 2010. Prodigal: prokaryotic gene recognition and translation initiation site identification. BMC Bioinform 11:11. 10.1186/1471-2105-11-119 PMC2848648 20211023

[81] El-Gebali S, Mistry J, Bateman A, Eddy SR, Luciani A, Potter SC, Qureshi M, Richardson LJ, Salazar GA, Smart A, Sonnhammer ELL, Hirsh L, Paladin L, Piovesan D, Tosatto SCE, Finn RD. 2019. The Pfam protein families database in 2019. Nucleic Acids Res 47:D427–D432. doi:10.1093/nar/gky995. 30357350 PMC6324024

[82] Buchfink B, Xie C, Huson DH. 2015. Fast and sensitive protein alignment using DIAMOND. Nat Methods 12:59–60. doi:10.1038/nmeth.3176. 25402007

[83] Arkhipova K, Skvortsov T, Quinn JP, McGrath JW, Allen CC, Dutilh BE, McElarney Y, Kulakov LA. 2018. Temporal dynamics of uncultured viruses: a new dimension in viral diversity. ISME J 12:199–211. doi:10.1038/ismej.2017.157. 29027998 PMC5739013

