# Supplementary figures and images for: Spatiotemporal Dynamics of Coastal Viral Community Structure and Potential Biogeochemical Roles Affected by an Ulva prolifera Green Tide
Source: mSystems. 2023 Feb 23;8(2):e01211-22. doi: 10.1128/msystems.01211-22 (PMC10134843; doi:10.1128/msystems.01211-22)

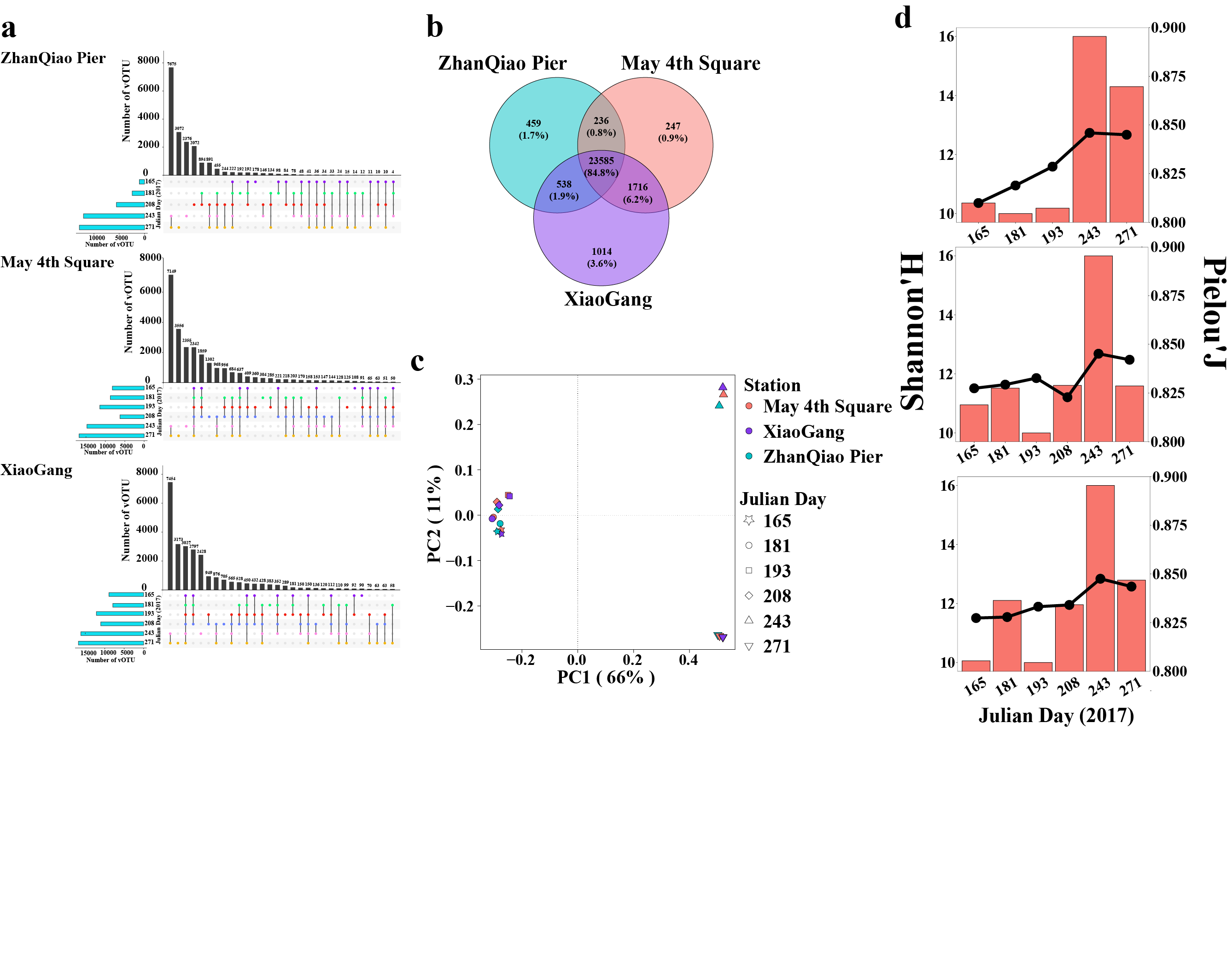

Supplement: FIG S1 [file msystems.01211-22-s0001.tif]

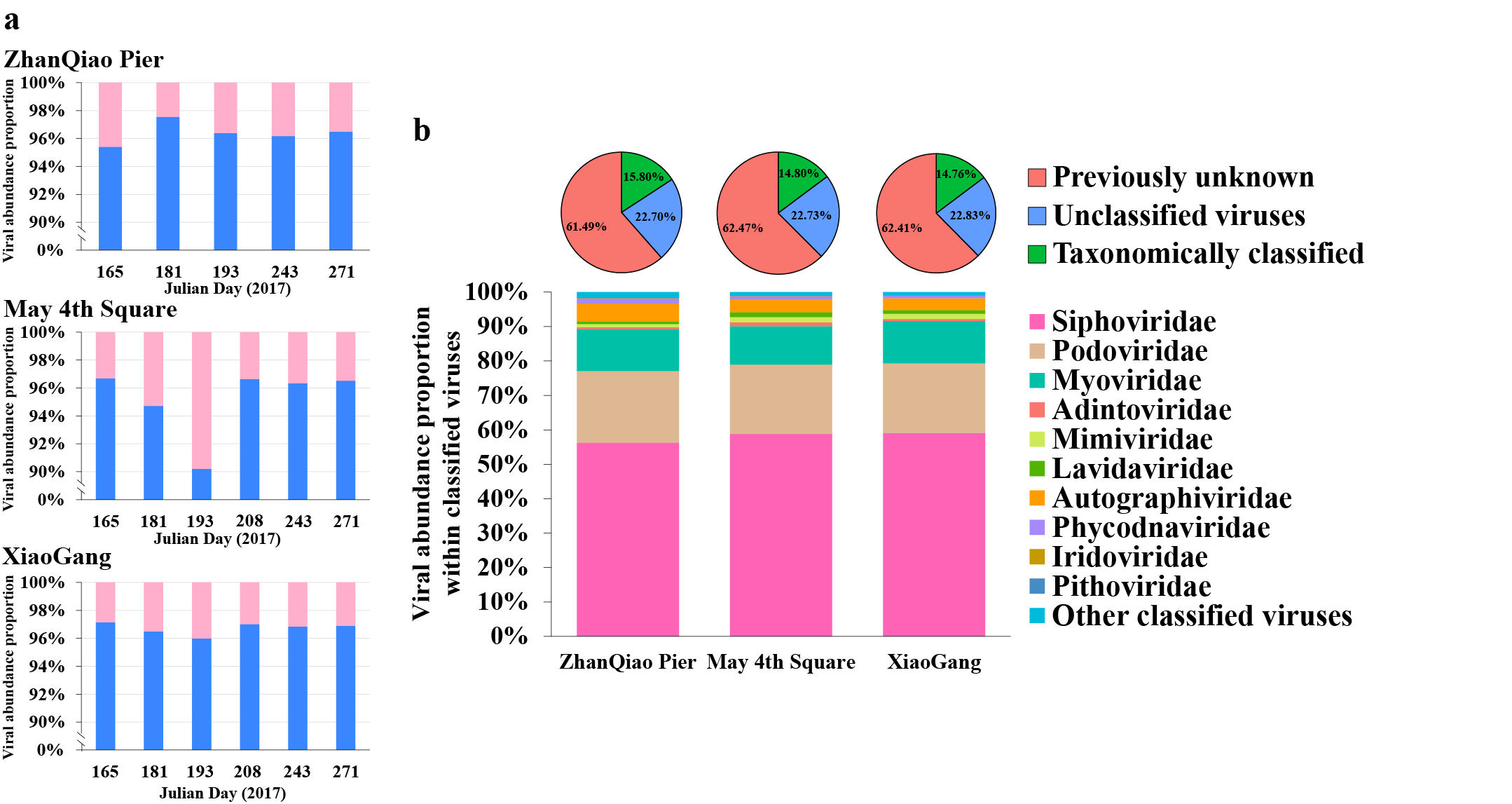

Supplement: FIG S2 [file msystems.01211-22-s0002.tif]

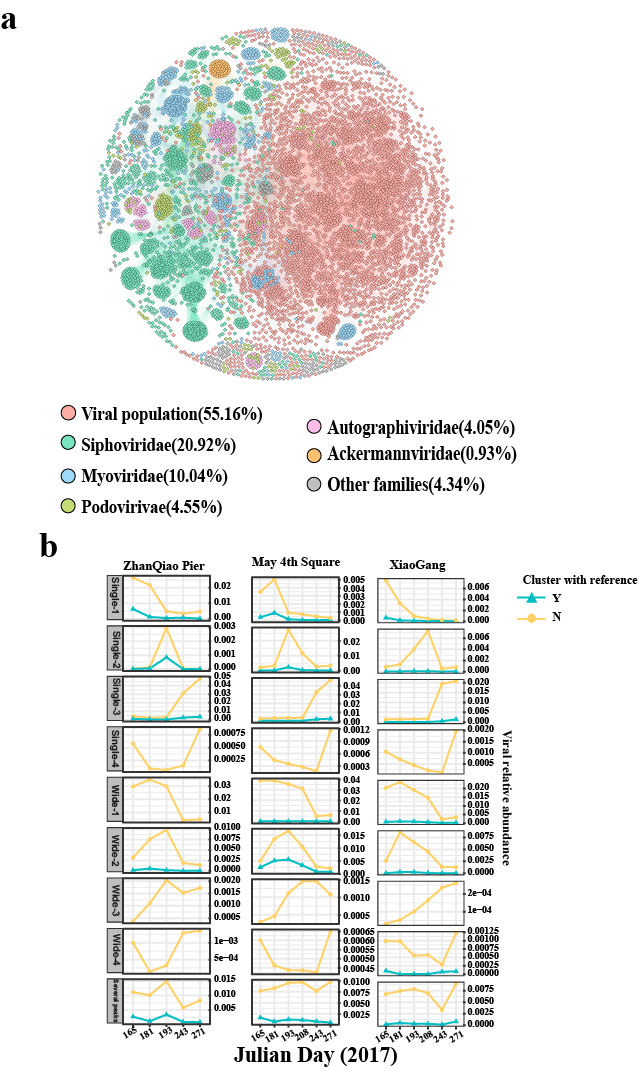

Supplement: FIG S3 [file msystems.01211-22-s0003.tif]

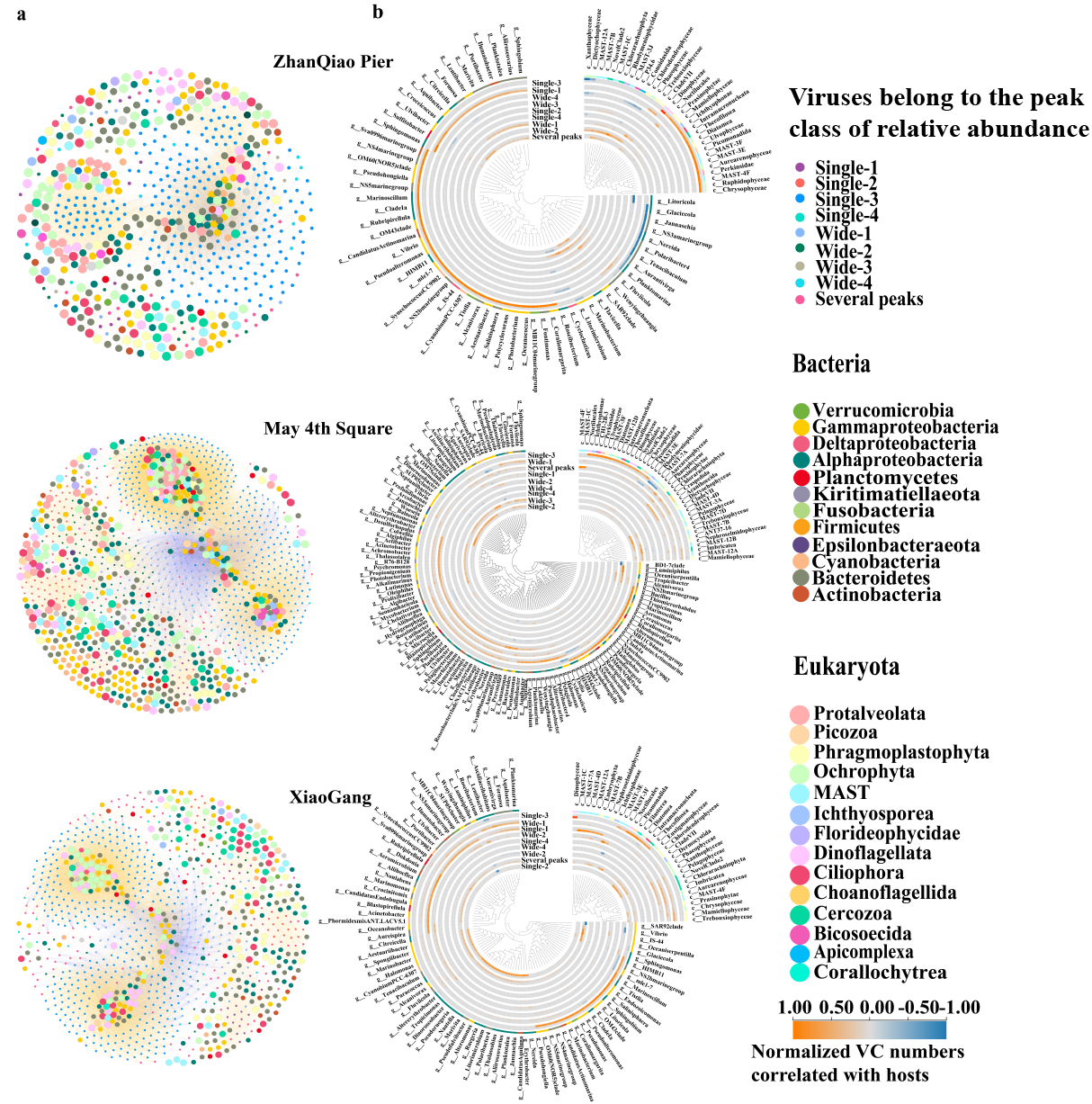

Supplement: FIG S4 [file msystems.01211-22-s0004.tif]

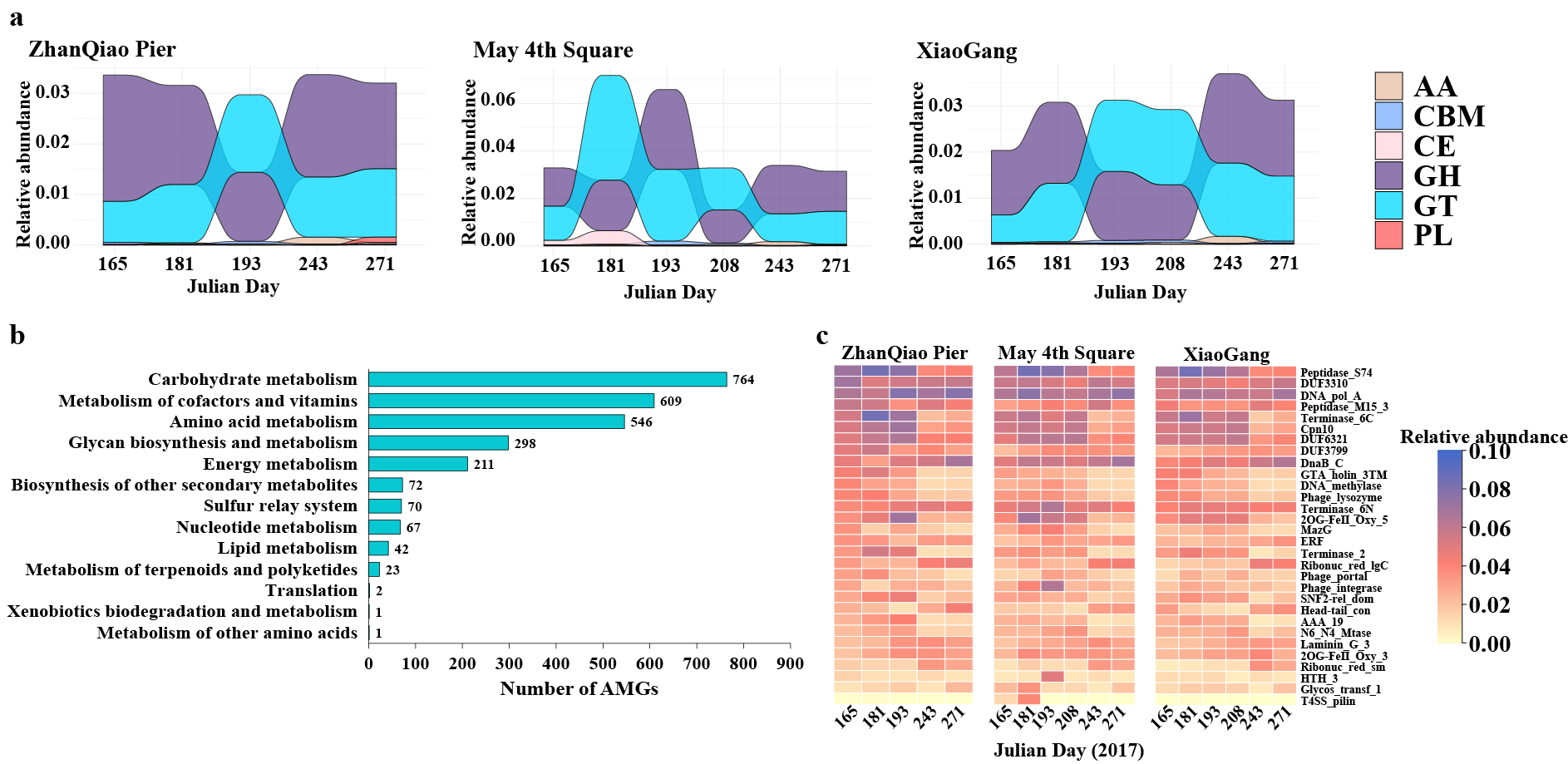

Supplement: FIG S5 [file msystems.01211-22-s0005.tif]
